# Effects of feed allowance and indispensable amino acid reduction on feed intake, growth performance and carcass characteristics of growing pigs

**DOI:** 10.1371/journal.pone.0195645

**Published:** 2018-04-05

**Authors:** Stefano Schiavon, Mirco Dalla Bona, Giuseppe Carcò, Luca Carraro, Lutz Bunger, Luigi Gallo

**Affiliations:** 1 Department of Agronomy, Food, Natural Resources, Animals and Environment, University of Padova, Legnaro, Padova, Italy; 2 Scotland’s Rural College (SRUC), Easter Bush, Edinburgh, United Kingdom; National Institute for Agronomic Research, FRANCE

## Abstract

The hypothesis that pigs placed on diets with reduced indispensable amino acid (AA) content attempts to offset the reduction in the nutrient density with increased feed intake was tested. In the experiment, feeds with a high or a low AA content were administrated to pigs fed *ad-libitum* or restrictively according to a 2 × 2 factorial design. Ninety-six barrows were housed in 8 pens (12 pigs/pen) equipped with automatic feeders. Within pen, and from 47 body weight (BW) onwards, 6 pigs were fed *ad libitum*. The others pigs were allowed to consume, as a maximum, the feed amounts indicated by the breeding company feeding plane to optimize the feed efficiency. In early (86–118 kg BW) and late (118–145 kg BW) finishing, the pigs of 4 pens received feeds with high indispensable AA contents (8.1 and 7.5 g lysine/kg in the two periods, respectively). The other pigs received feeds with reduced indispensable AA contents (lysine, methionine, threonine and tryptophan) by 9 and 18% in early and late finishing, respectively. Body lipid and protein (Pr) retentions were estimated from BW and back-fat depth measures recorded at the beginning and the end of each period. Nitrogen excretion was estimated as actual intake minus estimated N-retention (Pr/6.25). Pigs were slaughtered at 144 kg BW. Restricted feeding decreased feed intake (-7%), daily gain (-5%), carcass weight (-2.6%) and back-fat depth (-8.0%) but increased gain:feed ratio (+2%). The AA restriction increased feed intake (+5.9%), carcass weight (+4.9%) and intramuscular fat (+17.6%), and reduced carcass weight variation (-36%), with no effects on the feed efficiency and the estimated Pr (142 g/d). N excreted was reduced by feed (-9%) and dietary AA (-15%) restrictions. Irrespectively of the feeding level, the pigs responded to a reduction of the dietary essential AA content by increasing their feed intake.

## Introduction

Feed intake of an animal is influenced by its genotype (incl. sex, breed), health status, diet characteristics, various environmental factors and possible interactions. Several authors suggested that, under not limiting conditions, an animal will attempt to consume the amount of feed that will satisfy its requirement for energy and nutrients, according to the principle of the first limiting resource [1]. This proposition implies that a dilution of the dietary content of energy or nutrients may encourage the animal to increase its feed intake. The achievement of a feed intake reflecting the requirement for the deficient nutrients will depend on the ability of the pig to cope with various productive circumstances [2]. Therefore, mathematical models to predict feed and water intake and growth from information about the pig genotype, the feed characteristics, the environment conditions and the initial body status have been developed on the basis of these assumptions [3–5].

In farm animals, the interest in using low protein diets to reduce N excretion and emission of volatile N compounds is increasing [6–8]. In the pig industry, the introduction of low impact strategies need to be evaluated considering the feeding costs, the qualitative and quantitative aspects of the production, and the environmental benefits. It can be hypothesized that a pig kept on a diet lowered in essential amino acids (AA) content might attempt to increase its feed intake to achieve its genetic potential for lean growth, or protein retention [2]. But this was not always the case [9, 10]. The increased feed intake might cause an extra amount of energy eaten, so that the pig might become fat, with consequent changes in carcass weight and value according to the payment schemes of the reference meat market [11].

Under *ad libitum* (AL) conditions, all the pigs in a group would increase their feed intake in response to a dietary nutrient dilution, according to their age and productive stage. In some fattening pig circumstances, a certain degree of feed control, or restriction, is practiced to increase the feed efficiency, and to improve carcass quality by reducing the fat content [1, 11, 12]. Under restricted feeding conditions, a dilution of the dietary nutrient density would stimulate the pigs with the lowest voluntary feed intake to increase the consumption, where the pigs with the highest desire for feed are forced to maintain the feed intake to the limit imposed by the feeder equipment. Automated feeding stations can be used for individual control of feed distribution, allowing for both *ad libitum* and restricted feeding of pigs reared in the same pen, and to evaluate possible feeding level × dietary AA interaction.

The aim of this experiment was to assess the influence of a reduction in dietary indispensable AA on feed intake, growth performance, and carcass and meat quality traits of fast growing pigs fed by automatic feeding stations according to an *ad libitum* or a restricted feeding regime.

## Materials and methods

All the experimental procedures involving animals were approved by the “Ethical Committee for the Care and Use of Experimental Animals” of the University of Padua (CEASA, Legnaro, Italy).

### Pigs and experimental design

The study involved 96 Topigs Talent × PIC barrows born within the same week. They arrived at the end of February and were slaughtered at the end of June, thereby avoiding hot ambient summer temperatures. The average temperature in the housing rooms ranged from 20 to 25°C, from the start to the end of the trial. At the start of the experiment, the pigs averaged 47.1 ± 3.3 kg body weight (BW). They were allotted to 8 pens, 12 pigs/pen. In each pen, 6 pigs were fed *ad libitum* (AL) while the others were given restricted amounts of feed throughout the entire growing phase. Each pig of the restricted feeding group was allowed to consume, as a maximum, the daily feed amounts indicated by the feeding plane for Topigs Talent barrows suggested by the breeding company [13], with minor modifications (Table 1). These amounts were chosen by the breeding company to optimize feed efficiency. This plane was formulated to prevent excessive feed consumption by the pigs with the highest appetite, which might be less efficient because of their propensity for fattening.

**Table 1:** Planned feed allowances (kg/d) for restricted fed pigs.

Week	Initial BW	Final BW	Feed allowance^1^	Feeding phase
1	34	40	1.85	Acclimation
2	40	46	2.05	“
3	46	52	2.15	Growing
4	52	59	2.25	“
5	59	66	2.35	“
6	66	72	2.45	“
7	72	79	2.55	“
8	79	86	2.65	“
9	86	93	2.75	Early finishing
10	93	99	2.80	“
11	99	106	2.80	“
12	106	112	2.80	“
13	112	118	2.80	“
14	118	124	2.80	Late finishing
15	124	130	2.80	“
16	130	136	2.80	“
17	136	141	2.80	“
18	141	147	2.80	“

^1^ The feed allowances are those suggested for Topigs Talent barrows, with some modification [13].

From 87 kg BW onwards, the pigs in 4 pens received feeds with high indispensable AA contents (HAA) [14], while the others received feeds low in indispensable AA (LAA).

Individual BW was measured weekly using electronic scales and, from 87 kg BW onwards, back-fat depth (BF) was measured every two weeks with an A-mode ultrasonic device (Renco Lean-Meater series 12, Renco Corporation, Minneapolis, USA). The BF measure was taken above the last rib at approximately 5.5–8.0 cm from the midline, the distance increasing with increasing BW [15]. During the experiment, 4 pigs died or were discarded because of injuries (1 pig in the AL-LAA, 2 pigs in the RF-HAA, and 1 in the RF-LAA group). The corresponding data were removed so that the final dataset was drawn from 92 pigs.

### Feed distribution and control

Eight automated feeding stations (Compident Pig–MLP, Schauer Agrotronic, Austria), one per pen, were used to provide pigs with the designated amount of feed per day and to measure individual feed consumption. The pigs were allowed to visit the stations throughout the whole day. The stations were equipped with lateral barriers for limiting feed competition among pigs. When a pig visited the feeding station it was identified by an ear transponder, the automatic gate placed in front of the trough was opened and dry feed in form of pellets was released. The date and time of the feeding event, the time spent eating and the weights of feed eaten and leftovers were then recorded. The RF pigs had access to the station only if during their previous visit they had consumed less than the planned feed amount for that day [16]. The electronic feeder assigned the weight of eventual leftovers to the feed consumption of the pig of the following visit. As the feed was distributed by the station in doses of 200 g, some RF pigs may have been able to consume more than their planned feed amount (leftovers from the previous visit). Water was freely available from a nipple drinker placed in each pen, outside the feeding station.

### Feed formulation, manufacturing and chemical analysis

The commercial feeds used during acclimation (36–47 kg BW) and the growing (47–86 kg BW) periods provided, respectively, 10.1 and 9.9 MJ/kg of net energy (NE), 164 and 161 g/kg crude protein (CP), and 11.2 and 9.0 g/kg standardized ileal digestible lysine (SID Lys). The dietary indispensable AA content was not reduced during the growing phase.

The pigs in four pens received HAA feeds formulated to contain 9.8 MJ/kg of NE, 158 g/kg of CP and 8.1 g/kg of SID Lys in early finishing (87–118 kg BW) and 9.8 MJ/kg of NE, 155 g/kg of CP and 7.5 g/kg of SID Lys in late finishing (119–145 kg BW). The HAA diets provided the amount of the 4 main indispensable AA recommended for the genetic line, in slight excess than those recommended by the NRC [14]. The LAA diets administered from 87 kg onward were formulated from the corresponding HAA feeds by replacing soybean meal with corn and wheat grain (Table 2) to contain almost the same amount of NE (9.8 MJ/kg of NE), and by adding small amounts of crystalline AA to equalize the contents of four indispensable AAs (lysine, methionine, threonine, tryptophan) per CP unit of the feeds (Table 3). Thus, the proportions of the various indispensable amino acids, for HAA and the corresponding LAA feeds, were almost identical when expressed per unit of CP. The resulting LAA feeds contained 143 g/kg of CP and 7.3 g/kg of SID Lys in early finishing, and 126 g/kg CP and 6.0 g/kg of SID Lys in late finishing. Based on the average feed allowance per pig given in Table 1, in early (2.78 kg/d) and late finishing (2.80 kg/d), these concentrations provided 20.3 and 16.8 g/d of SID in the two periods, respectively.

**Table 2:** Ingredient composition (g/kg) of the diets used in the various phases of growth.

	Acclimation (35–47 kg BW)	Growing (47–86 kg BW)	Early finishing (86–118 kg BW)		Late finishing (118–145 kg BW)	
			High amino acid (HAA)	Low amino acid (LAA)	High amino acid (HAA)	Low amino acid (LAA)
Corn	405.5	442.3	465.7	465.4	416.8	448
Soybean meal	175	170	160	120	145	70
Wheat grain	160	160	160	200	180	220
Wheat bran	65	70	90	90	95	100
Wheat middling	60	100	80	80	120	120
Barley	60	0	0	0	0	0
Beef tallow and pig lard (1:1)	32	24	19	19	19	18
Calcium carbonate	13	13.5	14	14	13.5	13
Dicalcium phosphate	5	2	0	0	0	0
Sodium chloride	4.5	4.5	4.5	4.5	4.5	4.5
Vitamin and mineral premix^1^	2.5	2.5	2.5	2.5	2.5	2.5
L-Lys HCl	6.4	3.5	2.6	2.9	2.2	2.6
L-Thr	2.7	1.3	0.8	0.9	0.8	0.8
DL-Met	2.4	1.1	0.6	0.5	0.5	0.4
L-Trp	0.5	0	0	0	0	0
Choline HCl	0.5	0.3	0.3	0.3	0.2	0.2
Liquid organic acids	5	5	0	0	0	0

^1^Providing per kg of diet: 9000 UI of vitamin A, 2000 UI of vitamin D_3_, 1.5 mg of B_1_, 4mg of vitamin B_2_, 3 mg vitamin B_6_, 20 μg of vitamin B_12_, 30 mg of vitamin E, 2.1 mg of vitamin K_3_, 22.5 mg of pantothenic acid, 25 mg of niacin, 0.3 mg of folic acid, 0.3 mg of biotin, 50 mg of Mn, 113 mg of Zn, 125 mg of Fe, 17.5 mg of Cu, 1.75 mg of J, 0.375 mg of Se.

**Table 3:** Chemical composition (g/kg) and energy content (MJ/kg) of the diets used during the various phases of growth.

Item	Acclimation (35–47 kg BW)	Growing (47–86 kg BW)	Early finishing (86–118 kg BW)		Late finishing (118–145 kg BW)	
			High amino acid (HAA)	Low amino acid (LAA)	High amino acid (HAA)	Low amino acid (LAA)
Analyzed composition^1^						
Dry Matter	900	893	891	891	895	894
Crude Protein (N × 6.25)	163	163	159	141	161	133
Starch	423	387	440	454	421	454
NDF	107	120	111	111	123	130
Ether Extract	58	46	42	45	44	42
Ash	42	41	42	40	42	41
Lysine	12.5	10.3	9.3	8.5	8.8	7.2
Methionine	4.8	3.5	3.1	2.7	3.0	2.5
Threonine	8.4	7.0	6.3	5.9	6.4	5.1
Tryptophan	2.3	2.2	2.0	1.8	1.9	1.6
Calculated composition^2^						
Dry Matter	882	879	879	878	878	877
Metabolizable energy	13.6	13.5	13.4	13.4	13.3	13.3
Net energy	10.1	9.9	9.8	9.8	9.7	9.8
Crude Protein	164	161	158	143	155	126
Starch	428	435	442	462	440	478
Lipid	56	50	46	46	45	45
Linoleic acid	16	16	16	16	16	16
Ca	7.2	6.5	6.4	6.3	5.8	5.8
P	5.0	4.6	4.3	4.2	4.5	4.3
Available P	3.7	3.2	2.9	2.9	2.9	2.9
Lysine	12.3	10.0	9.2	8.4	8.6	7.0
SID Lysine^3^	11.2	9.0	8.1	7.3	7.5	6.0
Methionine	4.7	3.5	3.0	2.7	2.9	2.4
SID Methionine^3^	4.4	3.2	2.7	2.4	2.6	2.1
Threonine	8.2	6.9	6.3	5.8	6.2	5.0
SID Threonine^3^	7.3	5.9	5.4	4.9	5.2	4.2
Tryptophan	2.4	2.0	1.9	1.7	1.9	1.5
SID Tryptophan^3^	2.1	1.6	1.6	1.4	1.6	1.3

^1^Analitical results as a mean from 3 independent replications.

^2^ Computed from the ingredient composition according to NRC (2012).

^3^ SID: standardized ileal digestible amino acid content.

The feeds were produced from the same batches of ingredients. Based on actual prices in the market of reference, the cost of the LAA feeds was 8 and 15 euros/1000 kg lower than the corresponding HAA feeds in early and late finishing, respectively. Ten samples of each feed were collected on-line during feed manufacturing. The samples were pooled and mixed to obtain a 1-kg feed sample and independent sub-samples were taken. The sub-samples were analyzed (3 replicates) for dry matter (DM: # 934.01), N (# 976.05), ether extract (EE: # 920.29), ash (# 942.05) [17] and neutral detergent fiber inclusive of the contents of residual ash with amylase treatment (aNDF) [18]. Starch was determined after hydrolysis to glucose by liquid chromatography [19].

The amino acid content of the feed samples (0.5 g/sample) was determined according to the Council of Europe (chapter #2.2.56) [20]. Amino acids were released from the protein molecules by acid hydrolysis with HCl 6 M (Method 1) [20] at 110°C for 24 h. For cysteine/cystine and methionine, oxidation with performic acid was carried out before protein hydrolysis (Method 4) [20]. Tryptophan was determined by protein hydrolysis with Ba(OH)_2_ at 110° C for 20 h [21]. Pre-column derivatization was carried out using *o*-phthalaldeyde (OPA) for primary amino acids and 9-fluorenylmethyl chloroformate (FMOC-Cl) for secondary amino acids (Methods 5 and 7) [20]. The amino acids were separated and quantified using an HPLC (1260 Infinity, Agilent Technologies, Santa Clara, CA, USA). Separation was obtained on a Zorbax Eclipse-AAA (4.6 × 150 mm, 3.5 μm) operating at 40°C and a flow rate of 2 mL/min. The mobile phase consisted of 40 mM NaH_2_PO_4_ pH 7.8 (A) and acetonitrile:methanol:water (45:45:10, v/v) with gradient elution. A diode-array detector (DAD) and a fluorescence detector were used to detect amino acids with the following parameters: UV: 338 nm for OPA amino acids and 262 nm for the 9-fluorenylmethyloxycarbonyl (FMOC) amino acids; FLD: excitation wavelength/emission wavelength 266/305 nm. Amino acids were quantified following calibration using four standards ranging from 10 pmol/μL to 1 nmol/μL.

Dietary ME, crude protein, SID amino acids and other nutrients were computed from the actual ingredient composition of feeds and the tabular values for each ingredient [14]. Differences between analyzed and theoretical amino acid contents of the feeds were negligible.

### Body composition, energy and lysine utilization, and N balance estimates

Body composition, energy and lysine utilization, and N balance were estimated as described by Gallo et al. [16]. Briefly, empty BW (EBW) was estimated from BW at 86 and at 145 kg using the equation provided by Kloareg et al. [15] for barrows and gilts in the 85–150 kg BW range. Body lipid mass (BL, kg) was estimated from BF and BW [11]. Fat-free EBW mass (FFEBW) was computed as EBW minus BL. Body protein mass (BP, kg) was computed as 0.1353 × FFEBW^1.1175^, according to NRC [14].

Metabolizable energy intake (ME intake) was computed from the measured feed intake and dietary ME content. Metabolizable energy for growth (ME_g_) was computed assuming 44.35 MJ ME/kg for Pr and 52.30 MJ ME/kg for Lr [14]. The amount of ME used for maintenance (ME_m_) was computed as ME intake—ME_g_. The resulting value, expressed per unit of mean BW^0.60^, was compared with the maintenance requirement recommended by [14]. SID Lys intake was computed from feed intake and the dietary SID Lys content, while the SID Lys requirement was computed from feed intake, BW, maximum Pr and current Pr [14] with the equations 8–42 and 8–43. The results of the current experiment suggest a maximum of 167 g/d Pr for barrows.

Daily N excretion was determined as N intake—N retention, where N intake was calculated from feed intake and feed N content, and N retention was estimated from body Pr (Pr/6.25).

### Slaughter and carcass data collection

All pigs were slaughtered on the same day in one batch. The day before slaughter, the pigs were weighed, BF was measured and feed distribution stopped. After 14 h of fasting, the pigs were weighed again and then moved to the slaughterhouse, where they were slaughtered after a further 10 h of fasting and 2 h of resting at the slaughter-house. Pigs were stunned by a high concentration of carbon dioxide, and killed by cutting the jugular vein and exsanguination, according to the slaughter house standard procedures. Carcasses were scalded, de-haired, eviscerated and split down the midline according to commercial slaughtering procedures. Hot carcass weight was individually recorded and dressing percentage was computed. Carcass lean percentage [22–23] was computed from back-fat thickness and loin depth, which were measured on the left half of each carcass between the 3^rd^ and 4^th^ ribs 8 cm off the midline using a FOM (Fat-O-Meat’er, Carometec, Soeborg, Denmark).

Hot carcasses were processed according to a standard commercial procedure to obtain the main lean cuts (loin with ribs, neck with bones but without skin and subcutaneous tissues, shoulder with bones and skin, and ham) and fat primal cuts (back-fat with skin and belly), which were weighed separately. A sample of *longissimus lumborum* (LL) including the last two lumbar vertebrae was collected from the left loin of each of the 92 carcasses, placed in individual plastic bags, refrigerated for 24 h, then vacuum-packed at -20°C pending subsequent analyses. Thighs were deboned after 24 h of chilling and the deboned hams were weighed.

### Meat quality assessment

All the samples of LL were collected and thawed in vacuum-packaged bags for 24 h at 4°C, then removed from the packaging, blotted and weighed. Thawing losses were calculated to be the difference in weight between the fresh and thawed samples as a percentage of initial fresh weight. Cooking losses were determined on a 2.5 cm thick subsample of LL, which was weighed and sealed in a plastic bag, cooked in a water bath at 75° C for 50 min to reach a core temperature of 70° C, then cooled to room temperature, blotted and weighed. Cooking loss was calculated to be the difference between the pre- and post-cooked weights as a percentage of the pre-cooked weight. Shear force was measured on five cylindrical cores 1.00 cm in diameter from the same cooked sample sheared perpendicularly with a Lloyd® (Bognor Regis, UK) LS 5 series Warner-Bratzler shearing device (shearing speed 2 mm s^-1^) using the NEXIGEN Plus 3 software. The data from each sample were averaged before statistical analyses. Another subsample of LL was ground, mixed and homogenized for 10 *s* at 4500 *g* (Grindomix GM200; Retsch, Haan, Düsseldorf, Germany) and analyzed for moisture (#950.46), protein (#981.10), lipids (#991.36) and ash (#920.153) [17].

### Statistical analysis

The SAS MIXED procedure (SAS Inst. Inc., Cary, NC) was used to analyze the data according to the following linear model:

$$y_{ijkl}=\mu +FL_{i}+AA_{j}+FL\times AA_{ij}+pen{\left(AA\right)}_{k:i}+e_{ijkl};$$

where y_*ijkl*_ is the observed trait; *μ* is the overall intercept of the model; FL_i_ is the fixed effect of the *i*_*th*_ feeding level (*i* = 1, 2); AA_j_ is the fixed effect of the *j*_*th*_ kind of feed with different amino acid contents (*j* = 1, 2); pen_k:j_ is the random effect of the *k*:*j*_*th*_ pen within AA (*k* = 1, …, 8); FL × AA_ij_ is the effect of the interaction between feeding level and kind of feed; and e_*ijkl*_ is the random residual. Pen within AA and the residuals were independently and normally distributed with a mean of zero and variances of δ_*k*_^2^ and δ_e_^2^, respectively. In line with the experimental design, the effect of AA was tested using pen within AA as the error line, where the effect of FL was tested on the residual (animal) as error line, given that the pigs of both FL treatments were housed in the same pen. It is of note, that statistical analysis performed on estimated variables has to be considered with caution.

## Results

The feeding level × dietary AA density interaction was significant only exceptionally, thus the least square means for the main effects have been reported in the tables.

### Growth performance and feed efficiency

Feeding level affected performance in the growing and finishing periods (Table 4). Compared with pigs fed AL, RF pigs consumed less feed in the growing (*P* < 0.001), finishing periods (*P* = 0.002) and overall (*P* < 0.001), and grew 7% less in the finishing period (*P* = 0.033) and 5% overall (*P* = 0.014), but were more efficient in the growing period (*P* = 0.010) and overall (*P* = 0.050). They were also 3% lighter at the end of the study (*P* = 0.018) and exhibited less weight loss after 14 h of pre-slaughter fasting (*P* = 0.014).

**Table 4:** Growth performance of barrows fed *ad libitum* (AL) or restrictively (RF), feeds with high (HAA) or low (LAA) crude protein and indispensable AA contents^1^.

Item	Feeding level (FL)			Amino Acid level (AA)			*P values*		
	AL	RF	SEM	HAA	LAA	SEM	FL^2^	AA^3^	FL × AA
Body weight, kg:									
- Start of growing period^4^	47.0	47.2	0.49	46.7	47.6	0.49	0.78	0.22	0.81
- Start of finishing period^4^	86.0	85.3	0.90	84.9	86.3	1.06	0.48	0.41	0.85
- End of trial	145.8	141.2	1.35	140.4	146.6	1.35	0.018	0.018	0.82
- Weight loss for 14 h of fasting	3.85	2.88	0.28	3.37	3.36	0.28	0.014	0.98	0.67
Growth rate, kg/d:									
- Growing period	1.112	1.086	0.23	1.093	1.105	0.030	0.12	0.79	0.48
- Finishing period	0.881	0.823	0.020	0.817	0.887	0.020	0.033	0.038	0.77
- Overall	0.959	0.912	0.010	0.910	0.962	0.010	0.014	0.033	0.89
Feed intake, kg/d:									
- growing period	2.471	2.345	0.040	2.379	2.437	0.050	< 0.001	0.41	0.48
- finishing period	2.841	2.615	0.050	2.630	2.825	0.050	0.002	0.031	0.49
- overall	2.715	2.524	0.030	2.545	2.695	0.030	< 0.001	0.020	0.40
Actual feed intake–planned restricted feed allowance, kg/d^5^									
- growing period	0.121	-0.005	0.040	0.029	0.087	0.050	< 0.001	0.41	0.48
- finishing period	0.061	-0.165	0.050	-0.149	0.046	0.050	0.002	0.031	0.49
- overall	0.080	-0.111	0.030	-0.090	0.059	0.030	<0.001	0.020	0.40
Gain: feed:									
- growing period	0.451	0.463	0.005	0.460	0.454	0.005	0.010	0.52	0.94
- finishing period	0.307	0.311	0.003	0.306	0.312	0.003	0.49	0.28	0.050
- overall	0.352	0.359	0.003	0.355	0.356	0.003	0.050	0.93	0.09
Backfat thickness (P2)^6^, mm:									
- start of finishing period	9.2	9.2	0.22	9.2	9.2	0.22	0.96	0.93	0.46
- End of trial	13.7	13.2	0.40	13.0	13.8	0.43	0.34	0.23	0.26

^1^ Each data is the mean of 92 observations.

^2^ FL is the within pen effect of restriction (RF) compared to the *ad libitum* (AL) feeding.

^3^ AA is the effect of the dietary CP and AA content of the feeds.

^4^ During the growing period (47–86 kg BW) all pigs received feeds with the same protein and amino acids content. In the following growing-finishing period (86–145 kg BW) feeds with different content of crude protein and amino acids were used.

^5^ The planned restricted feed allowance is that suggested for Topigs Talent barrows [13], with minor modifications.

^6^ Measurements of ultrasound backfat thickness were collect from 86 kg BW onward.

Dietary AA content affected performance in the finishing period only, given that pigs were fed the same feed in the growing phase. Regardless of the feeding level, in the finishing period pigs on the LAA diets ate 7% more feed (*P* = 0.031) and grew 9% faster (*P* = 0.038) than pigs on the HAA diets. Greater feed intake and better growth rate of pigs fed LAA diets during finishing period resulted also in greater feed intake and growth rate in the whole trial (P < 0.05).

The difference between actual feed intake and planned feed allowance of pigs fed AL averaged -15 g/d with HAA and 175 g/d with LAA, whereas the differences were -166 g/d (HAA) and -56 g/d (LAA) for RF-fed pigs. Thus, regardless of whether pigs were fed AL or RF, feed intake was higher with LAA than with HAA contents, the growth rate of pigs on LAA diets was greater than that of pigs on HAA diets, and this resulted in a heavier BW at the end of the trial (+4%, *P* = 0.018).

**Fig 1:**
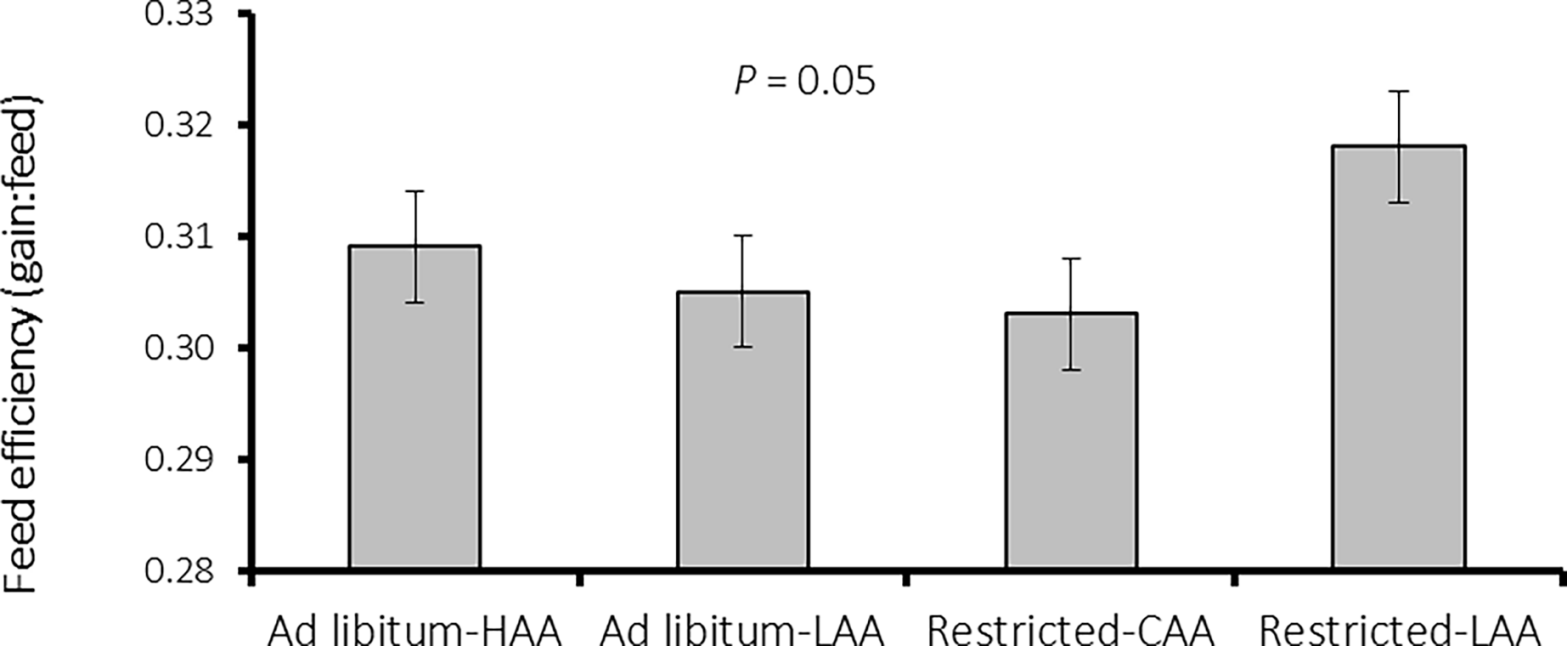
Influence of the interaction between feeding level (*ad libitum* or restricted) and dietary AA density [high amino acids feeds (HAA); low amino acids feeds (LAA)] on feed efficiency (gain:feed) of finishing pigs (118 to 145 kg of body weight; *P* = 0.05). Each bar is the least-squares mean from 23 observations and vertical bars indicate SEM.

Gain:feed ratio during the finishing period was the only trait for which feeding level interacted with dietary AA content (*P* = 0.050; Fig 1), as the feed efficiency of pigs on the LAA diet was better than that of pigs on the HAA diet only when fed RF, but not when fed AL.

### Estimated body composition, energy and SID lysine utilization

Estimated lipid (Lr) and protein (Pr) retentions were in the order of 257 and 142 g/d, respectively, with RF pigs having nearly 10% and 5% lower Lr (*P* = 0.13) and Pr (*P* = 0.050) than pigs fed AL, respectively (Table 5). The ME requirement for growth, taken as the sum of the ME requirements for Lr and Pr, was reduced with RF (-9%, *P* = 0.031) and increased as a consequence of the reduction in dietary AA (+11%, *P* = 0.043). The trend was similar to that observed for ME intake. The estimated ME required for maintenance was close to 1.00 MJ/kg BW^0.60^ for pigs fed AL, and 0.95 MJ/kg BW^0.60^ for RF pigs, with a significant effect of feeding level (*P* = 0.016) but not of AA content of the feeds.

**Table 5:** Estimated body composition, energy, lysine and N balance of barrows fed *ad libitum* (AL) or restrictively (RF), feeds with high (HAA) or low (LAA) crude protein and indispensable amino acid contents^1^.

Item	Feeding level (FL)			Amino acid level (AA)			*P values*		
	AL	RF	SEM	HAA	LAA	SEM	FL^2^	AA^3^	FL × AA
Mean metabolic weight (BW^0.60^)	16.0	15.4	0.29	15.6	15.9	0.34	0.05	0.58	0.09
Estimated body lipid^4^									
- initial, kg	16.0	15.9	0.25	15.8	16.1	0.26	0.92	0.38	0.67
- final, kg	34.4	32.4	0.85	32.1	34.7	0.95	0.06	0.11	0.54
- lipid retention (Lr), g/d	270.5	242.6	10.0	240.5	272.5	10.0	0.13	0.09	0.31
Estimated body protein^5^									
- initial, kg	14.5	14.3	0.17	14.3	14.5	0.20	0.46	0.67	0.86
- final, kg	24.4	23.7	0.26	23.6	24.5	0.25	0.06	0.046	0.46
- protein retention (Pr), g/d	145.8	138.5	3.00	137.4	146.9	3.00	0.050	0.10	0.42
ME requirement for Lr and Pr^6^, MJ/d	20.6	18.8	0.65	18.7	20.8	0.65	0.031	0.043	0.65
ME intake, MJ/d	38.0	35.0	0.57	35.1	37.8	0.57	0.002	0.027	0.47
ME for maintenance^7^, MJ/kg BW^0,60^	1.00	0.95	0.02	0.97	0.98	0.02	0.016	0.66	0.46
SID lysine requirement ^8^, g/d	19.1	18.1	0.45	17.9	19.2	0.45	0.11	0.08	0.34
SID lysine intake (g/d)	20.5	18.9	0.36	20.3	19.1	0.36	0.002	0.06	0.63
SID lysine surplus ^9^, g/d	1.40	0.83	0.40	2.37	-0.15	0.43	0.26	0.006	0.10
N intake^10^, g/d	65.9	60.7	1.16	65.7	60.8	1.16	0.002	0.024	0.66
N retention^10^, g/d	23.3	22.2	0.54	22.0	23.5	0.54	0.13	0.09	0.31
N excretion^10^, g/d	42.5	38. 6	1.04	43.8	37.4	1.16	0.003	0.008	0.28

^1^ Each data is the mean of 92 observations.

^2^ FL is the within pen effect of the restriction (RF) compared to the *ad libitum* (AL) feeding.

^3^ Amino acid level is the effect of the dietary CP and AA content of the feeds.

^4^ Computed from the empty BW (EBW) and the ultrasound backfat thickness measured at P2 level [15].

^5^ Computed from the fat free empty BW (FFEBW) using relationships between body protein and body water and ash [14].

^6^ Metabolizable energy (ME) computed assuming a requirement of 44.4 and 52.3 MJ/kg of protein and lipid retained, respectively [14].

^7^ Metabolizable energy (ME) for maintenance computed as: (ME intake–ME requirement for growth)/average BW^0.60^ [14].

^8^ Calculated from BW, feed intake and protein retention according to NRC [14].

^9^ Standardized ileal digestible (SID) lysine intake–SID lysine requirement.

^10^ N intake was computed from feed intake and its N content, N retention was computed as estimated N retention/6.25, and N excretion as N intake- N retention.

The estimated SID Lys requirement for maintenance and growth averaged 18.6 g/d and was not influenced by RF or by the reduction in dietary AA content in the early and late finishing periods. Lysine intake was reduced by RF (*P* = 0.002), and there was a tendency toward a reduction caused by the dietary AA content (*P* = 0.06). Therefore, the pigs on the LAA feeds were able to offset the large reduction in the dietary Lys content by increasing their feed intake. As a consequence, the Lys consumed surplus to the estimated requirement was close to zero for pigs on LAA diets and in the order of 2.2 to 2.5 g/d for those on the CAA diets, values significantly different from zero (*P* = 0.006).

Feed restriction (*P* = 0.002) and the low AA dietary content (*P* = 0.024) reduced the amount of N intake, without affecting N retention. As a result, estimated N excretion was lowered by feeding restriction (-9%, *P* = 0.003) and by the reduction in dietary AA content (-15%, *P* = 0.008).

### Carcass and meat quality

The carcass weight of pigs fed restrictively tended to be lower than that of pigs fed AL (*P* = 0.06), and pigs on LAA diets had considerably heavier carcasses than pigs on HAA diets (*P* = 0.012) (Table 6). The coefficient of variation of carcass weight was lower in the case of pigs fed restrictively on LAA diets than of those on all the other treatments (4.5% vs. 7.0%, data not shown), probably as a consequence of the smaller daily variation in their feed intake as compared to that of other pigs (coefficients of variation: 17% vs. 26%, data not shown).

**Table 6:** Carcass and meat quality of barrows fed *ad libitum* (AL) or restrictively (RF) feeds with high (HAA) or low (LAA) crude protein and indispensable AA contents.

Item	Feeding level (FL)			Amino acid level (AA)			*P values*		
	AL	RF	SEM	HAA	LAA	SEM	FL^2^	AA^3^	FL × AA
Carcass weight, kg	116.5	113.6	1.09	112.3	117.8	1.09	0.06	0.012	0.86
Carcass yield, %	79.9	80.4	0.23	80.0	80.4	0.23	0.13	0.31	0.94
Backfat thickness^1^, mm	20.8	19.1	0.69	18.9	21.0	0.81	0.037	0.12	0.29
Loin depth^1^, mm	64.3	65.2	0.68	64.8	64.7	0.68	0.38	0.88	0.51
Lean percentage (FOM)^4^, %	56.4	57.3	0.39	57.4	56.3	0.44	0.06	0.12	0.40
Main untrimmed lean and fat cuts, kg:									
- loin with ribs	19.5	19.3	0.19	19.0	19.8	0.19	0.42	0.028	0.50
- neck	8.2	8.0	0.08	8.0	8.2	0.08	0.050	0.09	0.56
- shoulder	16.9	16.7	0.16	16.7	16.9	0.16	0.34	0.32	0.73
- ham	30.9	30.4	0.28	30.1	31.2	0.28	0.26	0.031	0.77
- deboned ham	19.2	19.1	0.17	18.8	19.4	0.17	0.61	0.050	0.60
- backfat	8.9	8.3	0.25	8.0	9.1	0.27	0.07	0.036	0.79
- belly	13.6	13.1	0.20	12.9	13.7	0.22	0.06	0.048	0.65
- total main lean cuts	75.5	74.3	0.64	73.8	76.0	0.64	0.17	0.047	0.85
- total main fat cuts	22.4	21.3	0.39	21.0	22.8	0.39	0.047	0.016	0.93
Yield of untrimmed lean and fat cuts, % of carcass:									
- total lean	64.8	65.5	0.36	65.7	64.6	0.43	0.13	0.10	0.97
- total fat	19.2	18.7	0.23	18.6	19.3	0.24	0.13	0.09	0.96
Yield of deboned ham, % of untrimmed ham	16.5	16.8	0.10	16.8	16.5	0.10	0.15	0.24	0.73
Longissimus lumborum (LL) muscle composition, %									
- moisture	70.8	70.9	0.15	71.1	70.6	0.16	0.42	0.06	0.17
- protein	23.5	23.6	0.10	23.6	23.5	0.10	0.63	0.28	0.19
- intramuscular fat	4.3	4.1	0.17	3.9	4.5	0.18	0.61	0.037	0.67
- ash	1.2	1.2	0.01	1.2	1.2	0.01	0.76	0.67	0.93
Water holding capacity of LL, %									
- thawing loss	10.5	10.4	0.42	10.6	10.3	0.42	0.06	0.60	0.55
- cooking loss	30.3	30.2	0.26	30.3	30.3	0.27	0.81	0.95	0.07
Warner-Bratzler shear force of LL, kg	2.3	2.2	0.08	2.3	2.2	0.10	0.86	0.23	0.96

^1^Assessed with a Fat-O-Meat’er between the third to fourth last ribs at 8 cm off the carcass midline.

^2^ FL is the within pen effect of the restriction (RF) compared to the *ad libitum* (AL) feeding.

^3^ AA is the effect of the indispensable AA content of the feed.

^4^ Calculated from backfat thickness and loin depth taken between the third to fourth last ribs at 8 cm off the carcass midline [22–23].

Barrows on RF had thinner back-fat than AL fed pigs (*P* = 0.037) and tended to yield carcasses with nearly 2% more lean meat (*P* = 0.06). Accordingly, the main fat cuts of RF fed pigs were nearly 5% lower in weight than those of AL fed pigs (*P* = 0.047). The AA contents of the diets also influenced the weight of some lean and fat cuts, but neither feeding level nor dietary AA contents affected lean and fat cuts when expressed as proportions of carcass weight.

The feeding level exerted no effect on meat quality traits, while chemical composition, water holding capacity and shear force were similar for LL samples from AL-fed and RF barrows. Conversely, the intramuscular fat content of the LL was greater in pigs on diets with lower AA contents than in pigs on HAA diets (+ 18%, *P* = 0.037), with no further effects of dietary AA content on physical meat traits. No differences in the water holding capacity due to the feeding treatments were observed. There was a tendency for greater thawing loss in AL compared to RF (*P* = 0.06), and a tendency for interaction between RF and AL supply in cooking losses (*P* = 0.07).

## Discussion

### Effect of feeding level on feed intake, carcass quality and N excretion

Pigs raised in commercial conditions are normally penned and fed in groups, while in experimental studies they are frequently penned and fed individually [24]. Penning conditions affect competition for feed and the pigs’ social interactions and stress levels. The feeding behavior and growth performance of pigs in pens may therefore differ according to whether they are fed in groups or individually. In the current experiment, the pigs were penned in groups, but they had individual access to the feeding stations. Caution must therefore be exercised in extending the present results to commercial conditions.

Our results provide evidence that the feed intake of pigs fed *ad libitum* on HAA diets was close to that expected from the theoretical feeding curve recommended by the breeding company, confirming the feed restriction applied was small. Nevertheless, the feed intake of barrows on restricted feeding was approximately 5% lower than that of barrows fed AL and showed also less variation (coefficients of variation: 19% vs 29%, respectively). This was because the treatment forced some of the pigs in the former group to consume less than their desired amount of feed over the course of the trial.

Across the world, many growing pigs are fed *ad libitum* through the fattening period up to slaughter, without a ration scale. The use of rationing scales is especially appropriate for pigs of unimproved genotypes or for those slaughtered at heavy weights, and/or where there is a desire for increased feed efficiency and lean carcasses [1]. Few comparisons have been made between pigs fed *ad libitum* or restrictively with pigs penned in groups. In the current experiment, moderate restriction of the feed allowance applied from 47 to 145 kg BW led to a 2% improvement in feed efficiency, but a 3.2% decrease in final BW and 2.6% decrease in carcass weight. The lower influence of RF on carcass weight than on final BW was, at least partially, due to the 25% lower gut fill of RF than AL fed pigs, as suggested by the notable differences in BW losses after 14 h of fasting. Feed restriction resulted in an 8% reduction in carcass back-fat thickness, consistent with the 5% reduction in the total amount of untrimmed carcass fat cuts, but it had no influence on the proportions of lean and fat primal cuts in the carcass. These results are in general agreement with previous studies [1, 25–26], which suggested that feed restriction would improve feed efficiency and increase the leanness of the carcass, but it might reduce growth rate compared with *ad libitum* feeding. Dalla Bona et al. [27] also reported that feed restriction reduced the feed intake, growth rate and carcass weight of pigs slaughtered at 145 kg BW, but it improved their feed efficiency compared to AL feeding. Therefore, despite the slight negative effect on growth rate, a mild feed restriction might be advisable in some production systems due to its positive effects on feed efficiency. Moreover, the carcass weights of pigs fed restrictively on LAA diets showed the lowest coefficient of variation, suggesting a greater uniformity of carcasses of pigs of this group, which may be relevant for pig production chains given the economic value of carcass uniformity [28–29].

Overall, the mild feed restriction applied in this experiment also caused a 9.4% reduction in N excretion, which is similar to the results by Schiavon et al. [30] in beef cattle. Such strong reduction of N excretion would have important consequences in terms of number of pigs and meat, which can be finished per unit of agricultural land in those areas where a fixed amount N/ha is stated by law. In relative terms, taking AL as comparison, the feed restriction would approximately increase by 7.0% the number of pigs produced per unit of land.

### Effect of amino acid restriction on feed intake

Pigs on the LAA diets ate more feed than those on the HAA diets, regardless of the feeding level, and only a few interactions between the two factors were found. Although the SID lysine contents of the HAA and LAA diets differed by 9% in the BW interval between 87 and 118 kg and by 18% in the 118 to 145 kg BW interval, lysine intake was similar for pigs on all treatments and met their estimated requirements. As a consequence, and in contrast to the reduction in carcass weight associated with feed restriction, the decrease in dietary AA contents was also associated with a 4.9% increase in carcass weight. The pigs responded to the decrease in the AA contents of the feeds by increasing their feed intake, even when kept on the RF diet. This was possible because many of the RF-LAA pigs were forced to consume more feed, so that their actual average feed intake was very close to the maximum planned feed allowance and was nearly 6% greater than that of the RF-HAA pigs. This would also explain why the feed intake and carcass weights of RF-LAA pigs had the lowest coefficients of variation of all treatments. In addition, feeding behavior data from the current experiment showed that the eating rate of pigs on the LAA diets tended to be higher than that of pigs on HAA (56.1 *vs* 49.3 g/min, respectively; *P* = 0.07), as well as the eating rate of the AL pigs was lower than that of the RF pigs (49.1 *vs* 56.3 g/min, respectively; *P* = 0.016) [31]. Despite the increased feed intake, the total feeding cost of the LAA treatment was still slightly lower compared to HAA (390 euros/1000 pigs), because of the lower costs of the LAA feeds.

The results of the current experiment are not consistent with data from some studies that found that voluntary feed intake decreased when pigs were placed on diets deficient in protein or indispensable AA, specifically tryptophan [6, 32, 33]. For example, Schiavon et al. [6] found that a notable reduction in dietary crude protein and indispensable AA did not alter feed intake, and decreased feed efficiency, but in slow growing pigs kept under a restricted feeding regime for the dry-cured ham production. However, the current experiment is consistent with several other studies that found mild deficiencies in protein, lysine or threonine to increase feed intake [10, 34–35]. To this regard, it would be considered that it is commonly accepted that a reduction in dietary NE content leads to an increase in feed intake to maintain a constant net energy intake [36]. The influence of nutrient deficiencies on intake remains controversial, although Kyriazakis et al. [37] clearly showed that pigs are able to control their protein intake when fed in different ways. These disagreements may be reconciled by assuming that an animal will eat sufficient feed to satisfy its genetically determined requirements for nutrients, specifically energy, although environmental and social factors relating to diet, climate, disease or housing may cause it to either increase or decrease feed intake from its potential, as proposed by several authors [3, 10, 38–39]. Ferguson and Gous [2], for example, suggested that pigs on a low-protein diet would increase their feed intake to maintain their genetic potential for protein growth until the point at which the animals can no longer compensate, and feed intake will decline. The extent of compensation would also depend on the amount of heat the pigs need to lose. When the temperature rises to 20 to 24°C, the need to dissipate heat makes pigs progressively less able to increase their feed intake [3–4, 40]. Therefore, the results of the current experiment are consistent with the idea that pigs respond to a reduction in dietary indispensable AA content by attempting to increase their feed intake. The success of these attempts would depend on the genetic, environmental and dietary constraints operating during the growth period. This issue requires further investigation.

### Effect of amino acid restriction on carcass quality and N excretion

The small effect on feed efficiency, back-fat thickness, the proportions of lean and fat cuts in the carcass, and meat quality traits found when diets moderately decreased in their AA content were fed is in general agreement with findings of others [33, 41–42]. The greater intramuscular fat content of the *longissimus lumborum* found in pigs on LAA than in pigs on HAA is consistent with the results of Wood et al. [43], who observed a 43% increase in the intramuscular fat content of the LL of pigs fed on low protein—low lysine diets compared with control feeds, and with the findings of Suárez-Belloch et al. [42]. Schiavon et al. [6] also found that a sub-optimal protein and AA supply altered some quality traits of dressed hams by increasing the subcutaneous fat cover as well as the marbling score, in heavy pigs destined to the dry-cured ham production. An increase in intramuscular fat content may improve the eating quality of the meat [43–45] and could represent a potential extra value when farmers are paid for intramuscular fat. This would be the case for medium-heavy pig systems oriented towards high quality cooked ham production in Italy [46].

In the current experiment, despite the 6% increase in feed intake, pigs on LAA diets evidenced a 7.5% lower N intake than those on HAA diets. As the estimated N retention was not influenced by the dietary AA level, the estimated N excretion was markedly lower (15%) in pigs fed LAA diets. Therefore, and in agreement with other authors, we suggest that the use of diets lowered in indispensable AA and in N contents would reduce N excretion but would have a small effect on weight gain and carcass characteristics [16, 41, 47].

## Conclusions

Results from this experiment suggest that fast growing pigs respond to a reduction in dietary indispensable AA content by increasing their feed intake, under both *ad libitum* and restricted feeding conditions. This result support the theory that feed intake would reflect the requirement for the deficient nutrients, depending by the ability of the pig to cope with the productive circumstances. Moreover, a mild feed restriction resulted in a decreased feed intake as well as in a slightly lower carcass weight, but also in greater feed efficiency and carcasses with thinner back-fat compared to the *ad libitum* feeding regime. Restriction of the dietary indispensable AA content does not necessarily result in reduced growth performance, as in the current experiment we found heavier final body and carcass weights, with an alteration of the carcass and meat quality. In addition, a reduction in the dietary CP, or N, content, alongside a reduction in AA contents, may be a useful strategy for reducing N excretion in pigs and for some lowering of the feeding costs, depending on the price of the protein sources and of the synthetic amino acids.

## Supporting information

S1 Table Statistical descriptive. Growth performance of the experimental pigs.
(DOCX)

S2 Table Statistical descriptive. Estimated body composition, energy, lysine and N balance of the experimental pigs.
(DOCX)

S3 Table Statistical descriptive. Carcass and meat quality of the experimental pigs.
(DOCX)
