# Supplementary material for: Effects of feed allowance and indispensable amino acid reduction on feed intake, growth performance and carcass characteristics of growing pigs
Source: PLoS One. 2018 Apr 5;13(4):e0195645. doi: 10.1371/journal.pone.0195645 (PMC5886589; doi:10.1371/journal.pone.0195645)
Supplement: S1 Table — Growth performance of the experimental pigs. (DOCX) [file pone.0195645.s001.docx]

S1 Table. Statistical descriptive. Growth performance of the experimental pigs.

|  | Mean | Standard deviation | Coefficient of variation (%) | Minimum | Maximum |
| --- | --- | --- | --- | --- | --- |
| Body weight, kg: |  |  |  |  |  |
| - Start of growing period^1^ | 47.1 | 3.30 | 7.0 | 40.4 | 53.9 |
| - Start of finishing period^1^ | 85.6 | 4.89 | 5.7 | 74.9 | 96.9 |
| - End of trial | 143.6 | 9.80 | 6.8 | 117.3 | 166.4 |
| - Weight loss for 14 h of fasting | 3.4 | 1.91 | 56.6 | -3.2 | 7.6 |
| Growth rate, kg/d: |  |  |  |  |  |
| - Growing period | 1.099 | 0.09 | 8.4 | 0.9 | 1.3 |
| - Finishing period | 0.852 | 0.13 | 15.6 | 0.4 | 1.1 |
| - Overall | 0.936 | 0.09 | 10.1 | 0.6 | 1.1 |
| Feed intake, kg/d: |  |  |  |  |  |
| - growing period | 2.409 | 0.20 | 8.2 | 1.8 | 3.0 |
| - finishing period | 2.730 | 0.36 | 13.1 | 1.7 | 3.6 |
| - overall | 2.621 | 0.26 | 9.7 | 2.0 | 3.4 |
| Actual feed intake - planned restricted feed allowance, kg/d^2^: |  |  |  |  |  |
| - growing period | 0.059 | 0.20 | - | -0.5 | 0.7 |
| - finishing period | -0.050 | 0.36 | - | -1.0 | 0.8 |
| - overall | -0.014 | 0.26 | - | -0.7 | 0.8 |
| Gain: feed: |  |  |  |  |  |
| - growing period | 0.457 | 0.02 | 5.4 | 0.4 | 0.5 |
| - finishing period | 0.309 | 0.02 | 7.6 | 0.2 | 0.4 |
| - overall | 0.355 | 0.02 | 5.3 | 0.3 | 0.4 |
| Backfat thickness (P2)^3^, mm: |  |  |  |  |  |
| - start of finishing period | 9.2 | 1.48 | 16.2 | 6.5 | 15.0 |
| - End of trial | 13.4 | 2.62 | 19.5 | 7.0 | 21.0 |

^1^ During the growing period (47-86 kg BW) all pigs received feeds with the same protein and amino acids content. In the following growing-finishing period (86 – 145 kg BW) feeds with different content of crude protein and amino acids were used.

^2^ The planned restricted feed allowance is that suggested for Topigs Talent barrows [13], with minor modifications.

^3^ Measurements of ultrasound backfat thickness were collect from 86 kg BW onward.
