# Supplementary material for: Effects of feed allowance and indispensable amino acid reduction on feed intake, growth performance and carcass characteristics of growing pigs
Source: PLoS One. 2018 Apr 5;13(4):e0195645. doi: 10.1371/journal.pone.0195645 (PMC5886589; doi:10.1371/journal.pone.0195645)
Supplement: S2 Table — Estimated body composition, energy, lysine and N balance of the experimental pigs. (DOCX) [file pone.0195645.s002.docx]

S2 Table. Statistical descriptive. Estimated body composition, energy, lysine and N balance of the experimental pigs.

|  | Mean | Standard deviation | Coefficient of variation (%) | Minimum | Maximum |  |
| --- | --- | --- | --- | --- | --- | --- |
| Mean metabolic weight (BW^0.60^) | 15.7 | 1.57 | 10.03 | 13.6 | 18.6 | |
| Estimated body lipid^1^ |  |  |  |  |  | |
| - initial, kg | 15.9 | 1.64 | 10.3 | 12.4 | 20.6 | |
| - final, kg | 33.4 | 5.28 | 15.8 | 20.2 | 47.4 | |
| - lipid retention (Lr), g/d | 256.5 | 70.4 | 27.4 | 64.0 | 426.2 | |
| Estimated body protein^2^ |  |  |  |  |  | |
| - initial, kg | 14.4 | 0.92 | 6.37 | 12.4 | 16.9 | |
| - final, kg | 24.1 | 1.76 | 7.29 | 20.8 | 29.2 | |
| - protein retention (Pr), g/d | 142.3 | 23.4 | 16.4 | 73.2 | 202.6 | |
| ME requirement for Lr and Pr^3^, MJ/d | 19.7 | 4.07 | 20.6 | 6.60 | 29.0 | |
| ME intake, MJ/d | 36.5 | 4.77 | 13.1 | 23.3 | 48.4 | |
| ME for maintenance^4^, MJ/kg BW^0,60^ | 1.0 | 0.11 | 11.7 | 0.64 | 1.24 | |
| SID lysine requirement^5^, g/d | 18.6 | 3.12 | 16.8 | 9.49 | 26.9 | |
| SID lysine intake (g/d) | 19.7 | 2.58 | 13.1 | 13.4 | 27.9 | |
| SID lysine surplus^6^, g/d | 1.10 | 2.79 | - | -6.66 | 8.27 | |
| N intake^7^, g/d | 63.3 | 8.52 | 13.5 | 43.4 | 90.5 | |
| N retention^7^, g/d | 22.8 | 3.74 | 16.4 | 11.7 | 32.4 | |
| N excretion^7^, g/d | 40.6 | 7.38 | 18.2 | 24.2 | 66.5 | |

^1^ Computed from the empty BW (EBW) and the ultrasound backfat thickness measured at P2 level [15].

^2^ Computed from the fat free empty BW (FFEBW) using relationships between body protein and body water and ash [14].

^3^ Metabolizable energy (ME) computed assuming a requirement of 44.4 and 52.3 MJ/kg of protein and lipid retained, respectively [14].

^4^ Metabolizable energy (ME) for maintenance computed as: (ME intake – ME requirement for growth)/average BW^0.60^ [14].

^5^ Calculated from BW, feed intake and protein retention according to NRC [14].

^6^ Standardised ileal digestible (SID) lysine intake – SID lysine requirement.

^7^ N intake was computed from feed intake and its N content, N retention was computed as estimated N retention/6.25, and N excretion as N intake- N retention.
