# Supplementary material for: Effects of feed allowance and indispensable amino acid reduction on feed intake, growth performance and carcass characteristics of growing pigs
Source: PLoS One. 2018 Apr 5;13(4):e0195645. doi: 10.1371/journal.pone.0195645 (PMC5886589; doi:10.1371/journal.pone.0195645)
Supplement: S3 Table — Carcass and meat quality of the experimental pigs. (DOCX) [file pone.0195645.s003.docx]

S3 Table. Statistical descriptive. Carcass and meat quality of the experimental pigs.

|  | Mean | Standard deviation | Coefficient of variation (%) | Minimum | Maximum |
| --- | --- | --- | --- | --- | --- |
| Carcass weight, kg | 115.1 | 7.93 | 6.9 | 87.7 | 133.8 |
| Carcass yield, % | 0.8 | 0.02 | 2.0 | 0.7 | 0.8 |
| Backfat thickness^1^, mm | 19.9 | 4.02 | 20.2 | 11.0 | 30.0 |
| Loin depth^1^, mm | 64.8 | 4.50 | 6.9 | 42.0 | 74.0 |
| Lean percentage (FOM)^2^, % | 56.8 | 2.35 | 4.1 | 50.3 | 62.9 |
| Main untrimmed lean and fat cuts, kg: |  |  |  |  |  |
| - loin with ribs | 19.4 | 1.30 | 6.7 | 15.3 | 22.3 |
| - neck | 8.1 | 0.59 | 7.3 | 6.9 | 10.0 |
| - shoulder | 16.8 | 1.10 | 6.6 | 15.0 | 20.4 |
| - ham | 30.7 | 1.96 | 6.4 | 24.3 | 34.9 |
| - deboned ham | 19.1 | 1.15 | 6.0 | 15.5 | 20.7 |
| - backfat | 8.6 | 1.66 | 19.4 | 3.5 | 12.2 |
| - belly | 13.3 | 1.36 | 10.2 | 8.9 | 16.5 |
| - total main lean cuts | 74.9 | 4.42 | 5.9 | 61.5 | 86.7 |
| - total main fat cuts | 21.9 | 2.79 | 12.7 | 12.4 | 28.6 |
| Yield of untrimmed lean and fat cuts, % of carcass: |  |  |  |  |  |
| - total lean | 65.2 | 2.11 | 3.2 | 59.5 | 70.5 |
| - total fat | 19.0 | 1.51 | 7.9 | 14.2 | 22.4 |
| Yield of deboned ham, % of untrimmed ham | 16.6 | 0.72 | 4.3 | 14.9 | 18.2 |
| Longissimus lumborum (LL) muscle composition, % |  |  |  |  |  |
| - moisture | 70.8 | 1.05 | 1.5 | 68.5 | 74.2 |
| - protein | 23.5 | 0.65 | 2.8 | 21.5 | 25.5 |
| - intramuscular fat | 4.2 | 1.18 | 28.1 | 2.0 | 7.1 |
| - ash | 1.2 | 0.04 | 3.2 | 1.1 | 1.3 |
| Water holding capacity of LL, % |  |  |  |  |  |
| - thawing loss | 10.5 | 2.82 | 27.0 | 4.0 | 17.5 |
| - cooking loss | 30.3 | 1.65 | 5.5 | 26.2 | 35.4 |
| Warner-Bratzler shear force of LL, kg | 2.3 | 0.38 | 16.9 | 1.4 | 3.1 |

^1^Assessed with a Fat-O-Meat’er between the third to fourth last ribs at 8 cm off the carcass midline.

^2^ Calculated from backfat thickness and loin depth taken between the third to fourth last ribs at 8 cm off the carcass midline [22-23].
